# SCOPE: Surveillance of COVID-19 in pregnancy- results of a multicentric ambispective case-control study on clinical presentation and maternal outcomes in India between April to November 2020

**DOI:** 10.1371/journal.pone.0272381

**Published:** 2023-03-06

**Authors:** Sunesh Kumar, Neerja Bhatla, K. Aparna Sharma, Ramesh Agarwal, Ankit Verma, Vanamail Perumal, Poonam Shiv Kumar, B. S. Garg, Shivaprasad S. Goudar, Yeshita V. Pujar, Avinash Kavi, Vanita Suri, Bharti Joshi, Asmita Rathore, Madhavi M. Gupta, Ajay Kumar, Manju Puri, Deepika Meena, Sushma Nangia, Renu Arora, Sumitra Bachani, Pratima Anand, Shikha Seth, Rakesh Gupta, Rohini Sehgal, Anubhuti Rana, Archana Kumari, Shainy P., Kasturi Donimath, Guruprasad Gowder, Lakshmi Kedar, Tushar Kar, Sujata Mishra, Chinmayi Joshi, Yogendra Kabade, Saroja Kamatar, Saumya Nanda, Vandana Mohapatra, Janki Vellanki, Sarita Agarwal, Aparna Wahane Borkar, Aruna Kumar, Shabana Sultan, Neha Gangane, Pushpa Chaudhary, Anoma Jayathilaka, Neena Raina, Priya Karna, For the SCOPE Study Group

**Affiliations:** 1 All India Institute of Medical Sciences, New Delhi, India; 2 Mahatma Gandhi Institute of Medical Sciences, Wardha, Maharashtra, India; 3 Belagavi Institute of Medical Sciences, Belagavi, Karnataka, India; 4 Post Graduate Institute of Medical Education and Research, Chandigarh, India; 5 Maulana Azad Medical College, New Delhi, India; 6 Lady Hardinge Medical Delhi, New Delhi, India; 7 Vardhaman Medical College and Safdarjung Hospital, New Delhi, India; 8 Government Institute of Medical Sciences, Noida, Uttar Pradesh, India; 9 Karnataka Institute of Medical Sciences, Hubli, Karnataka; 10 J J M Medical College, Davanagere, Karnataka; 11 S C B Medical College, Cuttack, Odisha, India; 12 Fakir Mohan Medical College, Balasore, Odisha, India; 13 Gandhi Medical College, Hyderabad, Telagana, India; 14 AIIMS, Raipur, Chhatisgarh, India; 15 Government Medical College, Akola, Maharashtra, India; 16 Government Medical College, Bhopal, Madhya Pradesh, India; 17 WHO, New Delhi, India; 18 WHO, SEAR, New Delhi, India; University of Nebraska Medical Center, UNITED STATES

## Abstract

**Objective:**

To determine the clinical manifestations, risk factors, treatment modalities and maternal outcomes in pregnant women with lab-confirmed COVID-19 and compare it with COVID-19 negative pregnant women in same age group.

**Design:**

Multicentric case-control study.

**Data sources:**

Ambispective primary data collection through paper-based forms from 20 tertiary care centres across India between April and November 2020.

**Study population:**

All pregnant women reporting to the centres with a lab-confirmed COVID-19 positive result matched with controls.

**Data quality:**

Dedicated research officers extracted hospital records, using modified WHO Case Record Forms (CRF) and verified for completeness and accuracy.

**Statistical analysis:**

Data converted to excel files and statistical analyses done using STATA 16 (StataCorp, TX, USA). Odds ratios (ORs) with 95% confidence intervals (CI) estimated using unconditional logistic regression.

**Results:**

A total of 76,264 women delivered across 20 centres during the study period. Data of 3723 COVID positive pregnant women and 3744 age-matched controls was analyzed. Of the positive cases 56·9% were asymptomatic. Antenatal complications like preeclampsia and abruptio placentae were seen more among the cases. Induction and caesarean delivery rates were also higher among Covid positive women. Pre-existing maternal co-morbidities increased need for supportive care. There were 34 maternal deaths out of the 3723(0.9%) positive mothers, while covid negative deaths reported from all the centres were 449 of 72,541 (0·6%).

**Conclusion:**

Covid-19 infection predisposed to adverse maternal outcomes in a large cohort of Covid positive pregnant women as compared to the negative controls.

## 1. Introduction

Coronavirus disease 2019 (COVID-19) caused by Severe Acute Respiratory Syndrome Coronavirus-2 (SARS-CoV-2) rapidly triggered a global health emergency. On 12 March 2020, WHO declared it as a pandemic [1, 2]. Even with several unknowns about the risk of fetal exposure to SARS-CoV-2 during pregnancy, embryogenesis and fetal development, pregnant women were generally considered vulnerable due to past infectious disease outbreak experiences, physiological changes and existing co-morbidities that occur in pregnancy [3, 4].

Nearly 1.3 billion people live in India, of which approximately 26% (328 million) are women of reproductive age (15–49) [5]. In March 2020, when India went into a national lockdown to curb the transmission of the COVID-19 pandemic [2], there were many unknowns regarding pregnant women and COVID-19 [6–8]. Before the pandemic, pregnancy related deaths or maternal mortality ratio (MMR) in India was at 113 (105–123) per 100,000 livebirths with a lifetime maternal mortality risk of 0.3%. With nearly 25 million annual deliveries, India has one of the largest population of pregnant women at any given time, who were at potential high-risk for COVID-19 and related adverse outcomes including mortality.

On 2^nd^ April 2020, the first case of a pregnant woman with COVID-19 lab-confirmed diagnosis delivered at All India Institute of Medical Sciences (AIIMS), New Delhi. With rising cases, limited testing capacity and restriction on travel, there was an urgent need to develop standard treatment protocols and triage criteria to mitigate the impact of COVID-19 on pregnant women. We needed to understand the clinical presentation, management, risk factors, severity, and associated morbidity and mortality with it for the mother-baby dyad.

To demonstrate the scale and scope of country level collaborative efforts and generate high-quality data to contribute to the global evidence on COVID-19 and pregnancy management, the Surveillance of Covid-19 in Pregnancy (SCOPE) study was set up. Over the next nine months (Apr-Nov 2020), SCOPE was conducted as an observational, facility-based multi-centric study in 20 high burden obstetric units across India with the objective to build capacity of providers in providing care and documenting that through standardized reporting and evidence generation on clinical characteristics and outcomes of COVID-19.

## 2. Materials and methods

SCOPE is an ambispective observational case-control study of pregnant women with a laboratory-confirmed diagnosis of COVID infection who were admitted to the facility between 2^nd^April 2020 and 30^th^ November 2020 and was tested positive for COVID-19 at the facility.

Each centre participating in the study had a Principal Investigator (PI) and two Co-PIs one each from Obstetrics and Neonatology who facilitated data collection with the help of a dedicated research associate. AIIMS, New Delhi served as the main WHO Collaborating Centre (WHO-CC) that coordinated the study with three other WHO-CCs Postgraduate Institute of Medical Education & Research (PGIMER) Chandigarh, Mahatma Gandhi Institute of Medical Sciences (MGIMS), Wardha and KLE Academy of Higher Education and Research’s Jawaharlal Nehru Medical College (KAHER-JNMC) Belagavi. Each WHO-CC had four to five other satellite centres based on geographic proximity, delivery load in the centre and voluntary consent for participation in the study. However, the geographical representation of the study hospitals were spread across 7 states and 1 union territory.

**Fig 1:**
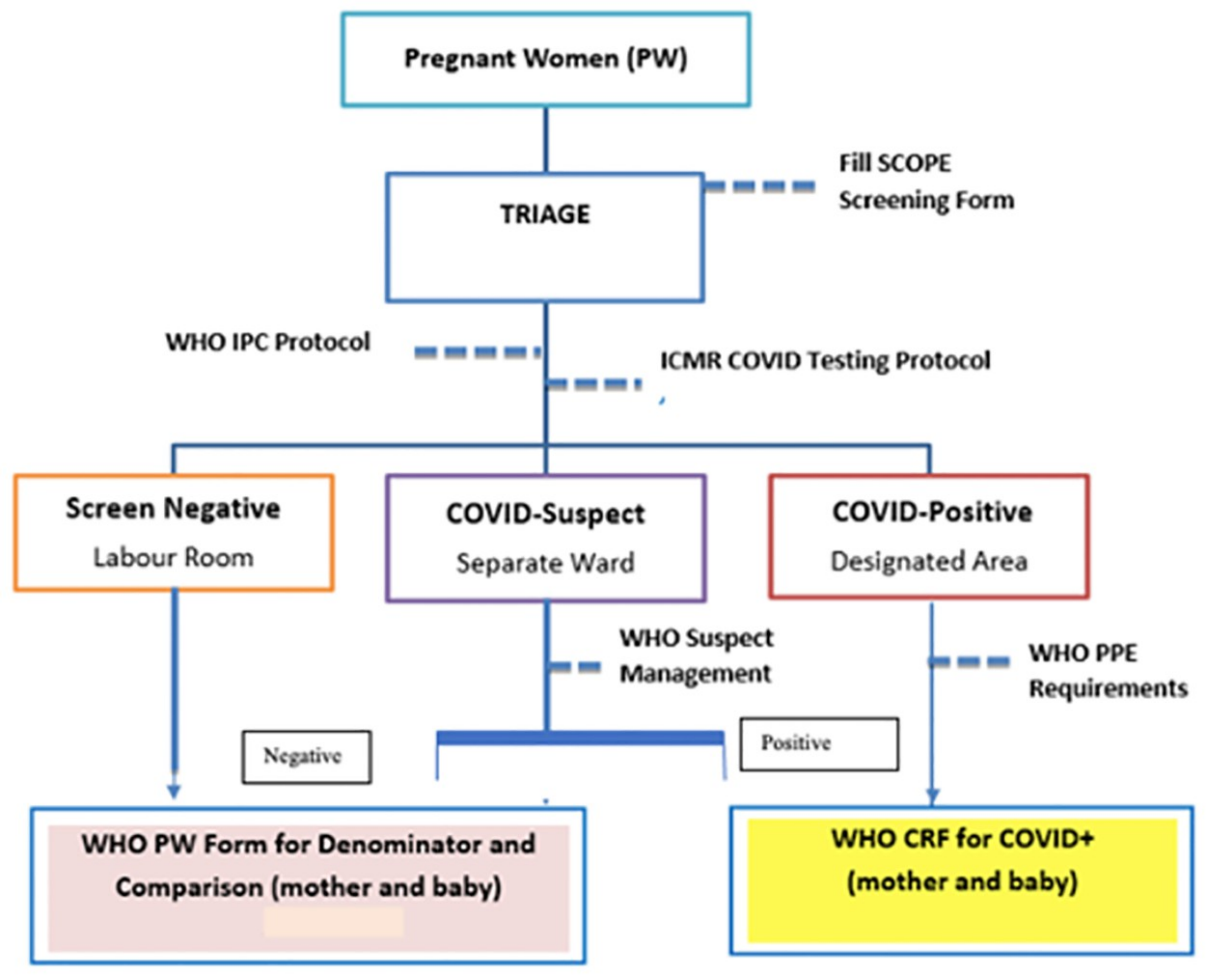
Patient recruitment flow chart.

Desk review of all existing reporting formats for all COVID-19 and pregnant women was done and a dedicated SCOPE reporting format was adapted from the global standardized WHO-CRF (case record form) for context-specificity and relevance for the Indian population while applying global standard tools. Orientation and capacity-building of all 21 centres was done in a standardized manner to harmonize data collection on the characteristics and outcomes of pregnant women admitted in these centres (Fig 1). Forms for collecting data among Covid negative women were created by the WHO-CC AIIMS, New Delhi which were reviewed and finalized following a consensus from all the participating teams.

**Fig 2:**
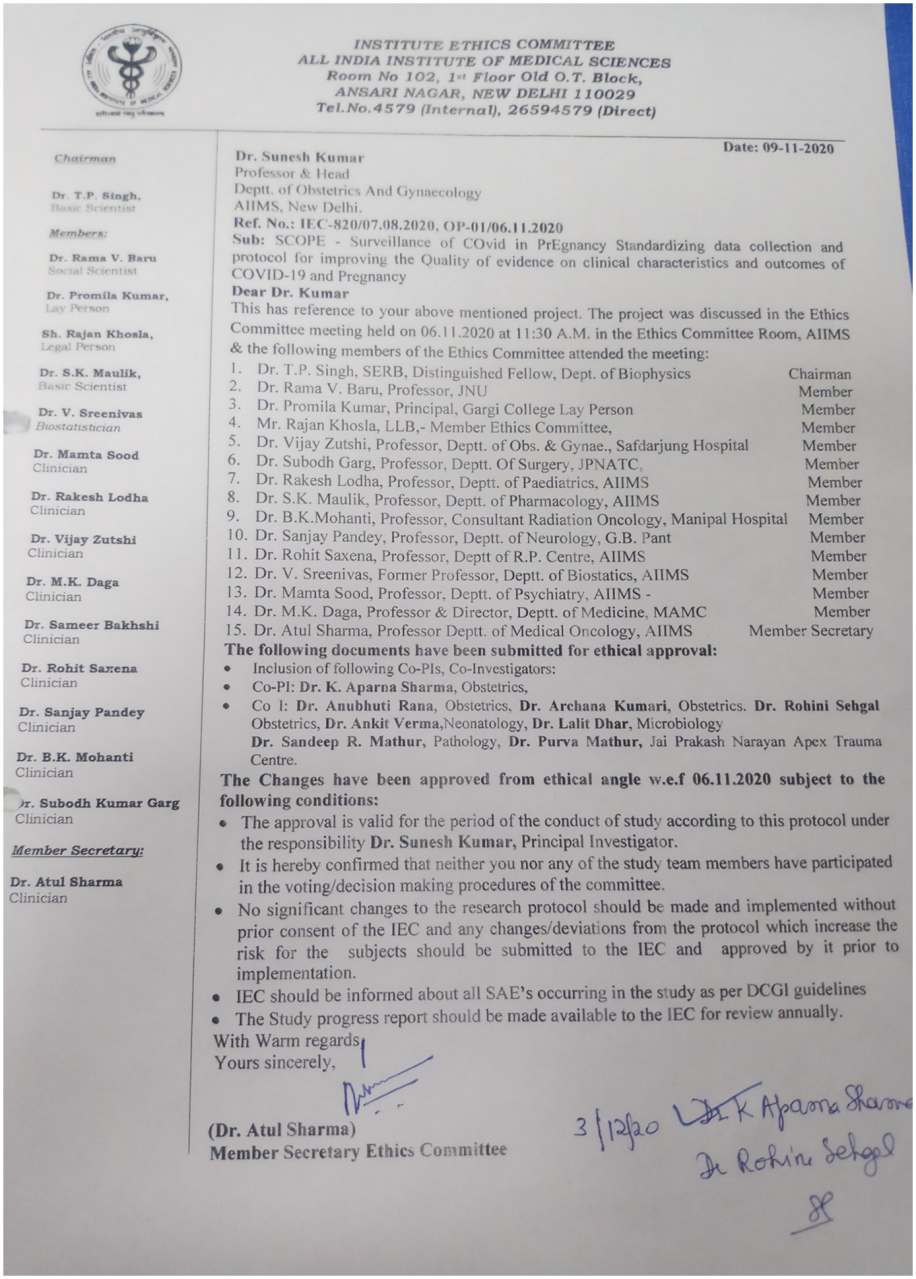
Ethics approval.

Each participating site received ethical clearance for the study from its institutional review board (Fig 2) and it was ensured that clinical management of the enrolled cases was not interfered with. Written informed consent was obtained from study participants for the prospective part and ethical waiver was obtained for the retrospective part as data was de-identified.

The inclusion criteria for cases -

- All COVID-19 positive pregnant women 18 years or older admitted at any stage of pregnancy, in labor or in postpartum period with laboratory confirmed diagnosis of COVID-19 by RT-PCR.

Inclusion criteria for controls

- Age matched COVID-19 negative pregnant women in 1:1 ratio from all pregnant women who tested negative for COVID -19 by RT-PCR and delivered during the study period were enrolled to create an unbiased sample.

Matching was done based on maternal age to improve study efficiency by improving precision as best as possible. Selection bias may be present as the entire target population could not be enrolled given the pandemic.

Detailed clinical characteristics for COVID positive pregnant women such as demographic characteristics, laboratory examinations where available, illness severity, and pregnancy outcomes for mother and new-born were recorded and compared between pregnant patients with COVID-19 or without COVID-19. Data regarding the pregnancy complications, disease severity, rates of maternal death, caesarean birth, stillbirths, neonatal infection, early neonatal death, and neonatal unit admission was also collected using the standard forms.

Several centres during the period of data collection were converted into dedicated COVID-19 centres and were able to enrol only cases. Controls for these cases were enrolled from within the 20 facilities included in the study to ensure data validity. Data was also collected on the total number of deliveries in the centres.

Quality control was done to ensure completeness, accuracy, and timeliness of data as per standard reporting formats and WHO normative data management guidelines. Dedicated research officers at each centre further reviewed hospital records and verified the data on all variables. Periodic online trainings were done with refresher trainings and problem-solving on using standard SCOPE forms to ensure that data was comparable and qualified for international comparison.

### 2.1. Statistical analysis

Data collected was converted to excel files and statistical analyses was done using STATA version 17·0 (Stata Corp, TX, USA). Categorical data were presented as frequency and percent values. Comparison of frequency data between the categories was carried out using Chi-square/Fisher’s exact test. Missing data for any variable were presented as the percentage of cases not available out of total. Odds ratios (ORs) with 95% confidence intervals (CI) were estimated using unconditional logistic regression.

Statistically, significant risk variables were discussed concerning clinical relevance. Continuous variables were tested for normality assumptions using the Kolmogorov-Smirnov test. Descriptive measures such as mean, standard deviation (SD) and range values were presented for normally distributed data. Testing of mean values was carried out by Student’s t-independent test/one-way analysis of variance test as appropriate. Skewed /non-normal data were presented as median and inter-quartile range (IQR) values. Comparison of median values was performed using the Wilcoxon rank-sum test/Kruskal Wallis test as appropriate.

Categorical data is presented as frequency and percent values. Comparison between the categories carried out using Chi-square/Fisher’s exact test. Overall missing data was under 2% across cases and controls. Odds ratios (ORs) with 95% confidence intervals (CI) estimated using unconditional logistic regression. Some degree of selection bias exists in the study.

Given the pandemic situation, sample size was governed by the disease incidence, thus formal power calculation was not done. The incidence of admission to hospital with confirmed COVID-19 infection in pregnancy was calculated and the denominators were collated from each centre. Univariable and multivariable logistic regression, adjusted for age, sex, comorbidities was done for comparing pregnancy outcomes. For all statistical inferences a two-sided probability of p<0·05 was statistically significant.

## 3. Results

During the study period a total of 76,264 pregnant women delivered across 20 centres during the study period. This includes reporting on 3723 pregnant women with a lab-confirmed COVID-19 positive report who were admitted to the centres at different gestational ages (Table 1) and included in the study.

**Table 1:** Distribution of gestation age among cases.

Gestation Age	N = 3723 (%)
First trimester (≤12 weeks)	72 (2·2%)
Second trimester (>12 weeks to ≤28 weeks)	192 (5·9%)
Third trimester (>28 weeks)	3000 (91·9%)
Missing data (incomplete)	459 (12·3%)

Age matched 3744 COVID-19 negative pregnant women out of the pregnant women who tested negative for COVID -19 by RT-PCR were recruited.

All centres were tertiary care facilities and as per Government of India (GoI) mandate, there was “no denial of services” for pregnant women that came to the health centre seeking care.

### 3.1. Maternal age

The average maternal (±SD) age of cases and controls was 26·2±4·4 years and 26·1±4·3 years respectively. The median parity in cases and control was one.

### 3.2. Gestation age among cases

Of 3723 COVID-19 positive pregnant women who were enrolled in the study, 3000 (91·9%) were in the third trimester, 192 (5·9%) were in the second trimester and 72 (2·2%) were in the first trimester. There was missing data on 459 (12·3%) as shown in Table 1 below.

### 3.3. Clinical presentation

Majority of the cases were asymptomatic (2115/3723,56·9%). Table 2 below shows the clinical manifestations at the time of hospital admission. There was less than 2% missing information on clinical presentation among cases. Of the controls, only 38 (0·9%) were symptomatic but tested negative. Among those who were symptomatic, (1605/3723, 43·1%), the table below shows the spectrum of symptoms.

**Table 2:** Spectrum of symptoms of COVID-19 among cases during pregnancy.

	N (%)
Fever	685 (18·7%)
Dry Cough	426(11·6%)
Malaise/Joint pain	192 (5·2%)
Runny nose	190 (5·2%)
Sore throat	181 (4·9%)
Cough with Sputum	174(4·8%)
Loss of Taste/Smell	135 (3·6%)
Nausea & Vomiting	135 (3·7%)
Shortness of breath	89 (2·4%)
Headache	52 (1·4%)

*Percentages calculated after subtracting the missing data

All the symptomatic cases were classified into mild, moderate, and severe disease according to ICMR-COVID-19 National Task Force/Joint monitoring Group (DteGHS) classification [9]. Based on this, 1506/1605(93.9%) had the mild disease; 67/1605(4.2%) had moderate disease and 32/1605(1.9%) had severe disease at the time of admission to the health facility.

### 3.4. Pre-existing comorbidities and risk factors

Out of 3723 cases, 277 (7·5%) women had pre-existing hypertension, 173 (4·7%) had pre-existing diabetes compared to 59 (1·6%) and 55 (1·5%) controls with pre-existing hypertension and diabetes respectively. Table 3 provides comparison of comorbidities in cases and controls.

**Table 3:** Co-morbidities among cases and controls.

Pre-existing Disease	Cases N = 3723 (%)	Controls N = 3744 (%)	P-value	Odds Ratio	(95% Confidence interval)
Chronic Hypertension	277 (7·5%)	59 (1·6%)	<0·001	4·98	(3·74–6·75)
Pre-existing Diabetes	173 (4·7%)	55 (1·5%)	<0·001	3·26	(2·38–4·51)

*Percentages calculated after subtracting the missing data

Anaemia is a severe public health problem among women in the reproductive age group and adolescent girls in India [10]. In our study, prevalence of anaemia (Hb<11g%) was 1696/ 3723 cases (50·1%);—mild anaemia (10–10·9gms%) in 830 (24·5%); moderate anaemia (7–9·9gms %) in 758 (22·5%) and severe anaemia (<7gms %) in 108 cases (3·2%).

### 3.5. Pregnancy-related complications and laboratory findings among cases and controls

Gestational diabetes, gestational hypertension and preeclampsia/eclampsia were significantly higher in cases compared to controls (Table 4). Other complications like placental abruption, antepartum and postpartum haemorrhage were also significantly higher among the cases.

**Table 4:** Pregnancy-related complications among cases vs controls.

Variable	Cases N = 3723 (%)	Controls N = 3744 (%)	P value	Odds Ratio	(95% Confidence interval)
Gestational Diabetes	131 (3·7%)	94 (2·6%)	0·007	1·4	(1·09–1·90)
Gestational Hypertension	284 (8·0%)	122 (3·3%)	<0·001	2·53	(2·02–3·16)
Preeclampsia/ Eclampsia	236 (6·6%)	146 (4·0%)	<0·001	1·69	(1·36–2·11)
Placental abruption	57 (1·6%)	19 (0·5%)	<0·0001	2·91	(1·7–5·2)
Postpartum Hemorrhage	66 (2·5%)	11 (0·3%)	<0·001	8·32	(4·35–17·5)

*Percentages calculated after subtracting the missing data

Additionally, thrombocytopenia was present in 776 cases (25·9%), thrombocytosis in 24 (0·8%) cases, leukocytosis (White blood cell count >16900 cells/cu.mm) in 173 (8·8%) cases and leukopenia (WBC <5600 cells/cu·mm) in 206/3015 (6·8%) cases. Data on D-Dimer levels, CRP and other inflammatory markers was not consistently available across all centres, hence not included.

### 3.6. COVID-19 treatment and management

In all participating centres, treatment was given as per institutional protocol at the time of study. Among cases, 3349 (92·0%) received antibiotics. Azithromycin was the most commonly prescribed antibiotic (56·9%). Antivirals were given to 159 (2·5%) in the form of Oseltamivir(n = 155), Favipravir (n = 2) and Remdesivir (n = 2), Antimalarials were given to 436 (12·0%). All received HCQ except 1 that received Artesunate; 419 (11·5%) received anticoagulation in the form of low molecular weight heparin. Corticosteroids were given to 203 (5·5%) women of which 106 women (2·9%) received steroids for achieving fetal lung maturity and 89 women (2·5%) received corticosteroids (Dexamethasone, Methylprednisolone and Hydrocortisone) for COVID-19 disease.

Table 5 below shows higher interventions required by trimester. It was also seen that 2.1% of primigravidas required oxygen therapy as compared to 3.1% of multigravidas. The corresponding numbers for ICU admission among both were 1.4% and 1.9% respectively while the requirement for invasive ventilation was 0.3% and 0.5% respectively. However, none of these were statistically significant.

**Table 5:** Trimester wise distribution of COVID-19 cases requiring additional support.

Cases (N = 3723)	First Trimester	Second Trimester	Third Trimester	Post Natal	Missing/ Loss to follow-up
Oxygen therapy (n = 100)	4 (4·1%)	3 (3·1%)	71 (72·4%)	20(20·4%)	2 (2·0%)
Intensive care unit (ICU) Admission (n = 65)	0 (0%)	2 (3·2%)	52 (83·9%)	8 (12·9%)	3 (4·6%)
Non-invasive ventilation (n = 12)	-	-	8 (66·6%)	4 (33·3%)	-
Invasive ventilation (n = 18)	1 (5·8%)	2 (11·8%)	11 (64·7%)	3 (17·6%)	1 (5·5%)

*Percentages calculated after subtracting the missing data

Further, among COVID-19 positive pregnant women (N = 3723), those with any pre-existing co-morbidities (n = 2125) were more likely to require additional intervention in the form of oxygen therapy and ICU admission (Table 6 below). Maternal deaths were also significantly higher among women with co-morbidities 32 (1·5%) when compared to women with no comorbidities 2 (0·1%) (p value <0·001) (OR = 12·2, 95% CI 3·1–105·6).

**Table 6:** Requirement of additional support in cases with or without co-morbidities.

Cases (N = 3723)	Comorbidities Present N = 2125 (%)	Comorbidities Absent N = 1598 (%)	P value	Odds Ratio	(95% Confidence interval)
Oxygen therapy (N = 100)	78 (3·7%)	22 (1·4%)	<0·001	2·73	(1·67–4·62)
Intensive care unit (ICU) Admission (N = 65)	51 (2·4%)	14 (0·9%)	<0·001	2·78	(1·51–5·46)
Non-invasive ventilation (N = 12)	11 (0·5%)	1 (0·06%)	0·015	8·31	(1·21–357·8)
Invasive ventilation (N = 18)	17 (0·8%)	1 (0·06%)	0·001	12·88	(2·01–538·47)

*Percentages calculated after subtracting the missing data

### 3.7. Pregnancy outcomes in cases versus controls

Of the total COVID-19 positive pregnant women (n = 3723), delivery data was available for 2949 (80%) and 3605 controls. Delivery outcomes were not available for women admitted with ectopic pregnancy, first or second trimester losses and in postpartum period. Table 7 shows the delivery outcomes in cases and controls.

**Table 7:** Delivery outcomes in cases vs controls.

		Cases	Controls	P value	Odds Ratio	(95% Confidence interval)
Onset of labour (N = 2949 cases. N = 2972 controls)	Spontaneous	1654 (56·0%)	1963 (66·6%)	<0·001	0·72	(0·63–0·82)
	Induced	665 (22·5%)	569 (19·4%)			
	Cesarean section before labour	550 (18·7%)	440 (14·8%)	<0·001	1·36	(1·19–1·57)
	Missing data	80 (2·7%)	772 (16·6%)	··	··	··
Mode of delivery (N = 2949 cases; N = 3605 controls)	Vaginal	1721 (58·4%)	2318 (64·3%)	<0·001	0·78	(0·70–0·86)
	Cesarean section	1228 (41·6%)	1287 (35·7%)	<0·001	1·29	(1·16–1·42)

*Percentages calculated after subtracting the missing data

There were a total 34 deaths among the covid positive women. Among these, 4 (11.8%) occurred in first trimester, 1 (2.9%) in the second trimester, 22 (64·7%) in the third trimester and 7 (20.6%) were post-natal or brought dead to the centres. The average age of women was 26.05 years. The mean period of gestation was 30.4 weeks. Twenty three out of 34 women (67.6%) required ICU stay, 27 women (79.4%) had co-existing anemia, 20 women (58.8%) had COVID related ARDS as the direct cause of death, 16 (47.05%) women had hypertension/pre-eclampsia, 2 (5.8%) women had postpartum hemorrhage and 2(5.8%) women had Sepsis.

Maternal deaths were also higher among those that had pre-existing co-morbidities (32 (1·5%) versus 2 (0·1%); p value <0·001; O.R = 12·2; CI (3·1–105·16). Details about causes of maternal deaths are listed (Supplement 1).

Details on ectopic and abortion were not available in the current study. A total of 3005 neonates (cases) were born to 3723 covid positive mothers during the study period(unpublished data). There were 86 (3·2%; 86/2718) stillbirths among cases while in controls there were 74 still births (2·1%; 74/3579) (p = 0·006)(OR = 1·55, 95% CI 1·11–2·15). Majority of the neonates in both cases (74·3%, n = 2177) and the control (75·2%, n = 2622) group were born at term gestation, and had normal birth weight, 70·8% (n = 1655) in cases and 71·1% (n = 2957) in the control group. Delayed cord clamping was done in 47% (n = 1340) of the cases as compared to 83·7% (n = 2895) of controls, p = 0·001. Skin to skin contact was significantly lower in the cases (36·5%, n = 1033) as compared to the controls (80·7%, n = 3345), p = 0·001. Neonates requiring resuscitation in cases were significantly higher than controls 4·8% (n = 138) versus 2·5% (n = 104), p = 0·001 respectively. Vaccination received at the time of birth was significantly lower in the cases (64·2%, n = 1736) as compared to the controls (95·51%, n = 3597), p = 0·001. Detailed neonatal outcomes including stillbirths from the SCOPE study will be published in a subsequent manuscript.

## Discussion

The SCOPE study gives a clear, insight into the clinical presentation, management, and outcomes among COVID positive pregnant women as compared with age matched COVID negative controls. This is the single largest data set collected in a standardised manner from any low-middle income country to date. While several challenges including a catastrophic second wave in India led to delays in timeliness, extensive effort was made across all centres to ensure data quality particularly completeness and accuracy.

We found that among COVID-19 positive cases, 56·9% women were asymptomatic and only 43·1% women were symptomatic. Overall, 40·4% had mild disease, 1·8% women had moderate disease, while 0·9% women had severe disease, consistent with other studies documenting the first wave across the world [11, 12]. In the review by Allotey et al, around three quarters (73%) of the 906 pregnant Covid positive women among the universal screening population were asymptomatic. The most common symptoms reported were fever (40%) and cough (41%) [12].

Jering et al reported that of the 6380 women with COVID-19, 98·9% were discharged in stable state, 3·3% required intensive care, 1·3% women needed mechanical ventilation, and 0·1% died in the hospital [11].

Additionally, we saw with certainty that pre-existing maternal co-morbidity increased the requirement of supportive care like oxygen support, ICU stay, and need for ventilatory support. Women with pre-existing co-morbidities must be considered as a high-risk group. Providers need to accelerate and make-up for service disruptions in antenatal care to manage women with pre-existing co-morbidities against unnecessary exposure to the virus. Innovative approaches such as virtual or online consultations could be leveraged as and when possible.

Previous studies have reported leukocytosis in 26% women, lymphopaenia in 33% and raised C reactive protein levels in 49% women [12]. Current study, anemia was seen in 50·1% women,8·8% women had leukocytosis, 25·9% women had thrombocytopenia, and 0·8% women had thrombocytosis. Anemia is widely prevalent in the country and the prevalence corresponds to the general prevalence among the pregnant population.

Caesarean section rate was significantly high in both cases (41·6%) and controls (35·7%) as has been previously reported in other studies. We, now know that COVID is not an indication for C-section however, with limited personal protective equipment (PPE) early on, fear among providers and clients as well as absence of clear guidelines may have contributed to the increased rates.

Pregnancy related maternal and neonatal complications are increased in women with COVID-19 than those without the disease. In our study, pregnancy complications of preeclampsia, antepartum haemorrhage and postpartum haemorrhage was also seen more among the cases. As reported by Jering et al study, Preeclampsia was seen in 8·8% of cases versus 6·8% of controls (p<0·001) (OR = 1·36, 95% CI 1·22–1·46) [11]. Pre-eclampsia has been reported to be associated with severe COVID-19 and increased mortality [11–13], but it requires further assessment as the clinical presentation of severe pre-eclampsia could mimic worsening COVID-19 [14].

A higher proportion of still births during this time in both cases and controls group also reflects the disruption in routine antenatal, transport and emergency care.

Metaanalysis conducted by Allotey et al showed stillbirths among 0·9% of cases and 0·5% of controls (OR = 2·84, 95% CI 1·25 to 6·45) [12].

Maternal death was the most significant outcome of interest. A total of 34 deaths occurred among the 3723 (0·9%) COVID positive women. Of these, Covid related ARDS was seen in 20 out of the 34 mothers (58.8%). while Covid infection was a contributory factor in others. Total maternal deaths in COVID negative women across all centres was 449 of 72,541 (0·6%) (p value = 0·020) (OR = 1·5,95% CI 1·03–2·15). Maternal mortality ratio among COVID-19 positive pregnant women was 913 per 100,000 livebirth vs 618 per 100,000 livebirths overall across all centres in the study period. Maternal death was reported in 8/1605 (0·50%) women overall in PAN-COVID, in 3/651 (0·46%) of those with confirmed infection and in 4/2399 (0·17%) AAP-SONPM registrants [13]. US premier healthcare database showed mortality of cases (0·1%) and no mortality in Covid negative women (p<0·001) [11]. Metanalysis by Allotey et al reported mortality in 0·7% of cases and 0·2% of controls. (OR = 2·85, 95% CI 1·08 to 7·52) [12].

WHO recommended delayed cord clamping, early skin to skin contact, Kangaroo mother care (KMC) and breastfeeding in the neonates born to COVID positive mothers [15–17]. We found that maternal SARS-CoV-2 infection had created a significant hinderance in ensuing the routine delivery room practices like delayed cord clamping (47% versus 83·7%), skin to skin contact (36·5% versus 80·7%) and early initiation of breast feeding (57·1% versus 90·1%) in the cases as compared to the controls respectively(unpublished data). Detailed discussion will be published in a subsequent manuscript.

In our study, overall outcomes were found to be concerning for both the mothers and neonates born to the COVID positive mothers. The key strength of the study is that it reflects the collaborative efforts of multiple centres across the country which came together within a short span of time to generate data on the effect of COVID in pregnancy. The geographical expanse of the centres across the country also makes the data more generalisable. While all efforts were made to remove duplicate data and avoid double counting of participants in evidence synthesis, there is a chance of human error. The main limitations remain that the comparable data from the second wave across the participating centres was not gathered to better understand the disease pattern. The study lessons were however leveraged to bring stakeholders together and introduce vaccinations among pregnant and lactating women in India.

SCOPE reiterates that during any emergency or future health emergency, the significance of preparedness cannot be over-emphasised. There is an absolute need to have standard protocols for pregnancy management for infectious diseases like COVID-19, vigorous facility-level triage system as well as capacity building support for providers. During the study period, significant efforts were made into training the providers in the participating centres but also disseminate this evidence-based information across the country.

## Supporting information

S1 Table COVID related maternal deaths with ICD-10 coding. (DOCX)

S1 File (XLSX)
