# Supplementary material for: SCOPE: Surveillance of COVID-19 in pregnancy- results of a multicentric ambispective case-control study on clinical presentation and maternal outcomes in India between April to November 2020
Source: PLoS One. 2023 Mar 6;18(3):e0272381. doi: 10.1371/journal.pone.0272381 (PMC9987819; doi:10.1371/journal.pone.0272381)
Supplement: S1 Table — (DOCX) [file pone.0272381.s001.docx]

S1 Table: COVID related maternal deaths with ICD-10 coding

| **S No** | **Age (years)** | **POG**  **(Weeks)** | **ICU stay** | **Delay in seeking care** | **Underlying cause** | **Contributory**  **Cause** |
| --- | --- | --- | --- | --- | --- | --- |
| 1 | 35 | 30 | Yes | No | Pre-eclampsia  (O14·1) | COVID infection  (U07·1)  Kidney disease  (O10·2) |
| 2 | 32 | 36 | Yes | No | ARDS  (O98·5,U07·1) | PPH  (O72·1)  Anemia  (O99·0) |
| 3 | 25 | 33 | No | Yes | Pre-eclampsia  (O14·1) | COVID infection  (U07·1)  Anemia  (O99·0) |
| 4 | 35 | 33 | Yes | No | ARDS  (O98·5,U07·1) | Pre-eclampsia  (O14·1)  Anemia  (O99·0) |
| 5 | 24 | PND-6 | Yes | No | PPH  (O72·1) | COVID infection  (U07·1)  Anemia  (O99·0)  Kidney disease  (O10·2) |
| 6 | 24 | 34 | Yes | Yes | ARDS  (O98·5,U07·1) | Pre-eclampsia  (O14·1) |
| 7 | 22 | PND-14 | No | No | ARDS  (O98·5,U07·1) | Pre-eclampsia  (O14·1)  Anemia  (O99·0) |
| 8 | 26 | 40 | Yes | Yes | Pulmonary embolism  (O88·2) | COVID infection  (U07·1)  Anemia  (O99·0) |
| 9 | 25 | 26 | Yes | Yes | ARDS  (O98·5,U07·1) | Asthma  (O99·5) |
| 10 | 26 | 36 | No | Yes | ARDS  (O98·5,U07·1) | Anemia  (O99·0) |
| 11 | 25 | 37 | No | No | Pulmonary embolism  (O88·2) | COVID infection  (U07·1) |
| 12 | 32 | 37 | No | Yes | ARDS  (O98·5,U07·1) | COVID infection  (U07·1)  Pre-eclampsia  (O14·1) |
| 13 | 30 | 31 | Yes | Yes | ARDS  (O98·5,U07·1) | COVID infection  (U07·1)  Anemia  (O99·0)  Diabetes (O24·9) |
| 14 | 25 | 34 | No | Yes | ARDS  (O98·5,U07·1) | Diabetes  (O24·9)  Hypertension  (O13) |
| 15 | 30 | PND 16 | Yes | Yes | Puerperal sepsis  (O85) | COVID infection  (U07·1)  Pre-eclampsia  (O14·1)  Anemia  (O99·0) |
| 16 | 35 | 30 | Yes | Yes | ARDS  (O98·5,U07·1) | Pre-eclampsia  (O14·1)  Anemia  (O99·0)  Kidney disease  (O10·2) |
| 17 | 35 | 36 | No | Yes | Pre-eclampsia  (O14·1) | COVID infection  (U07·1)  Anemia  (O99·0) |
| 18 | 26 | PND-1 | No | Yes | ARDS  (O98·5,U07·1) | Anemia  (O99·0) |
| 19 | 30 | 32 | No | Yes | DIC  (O99·1) | COVID infection  (U07·1)  Anemia  (O99·0) |
| 20 | 20 | 3 MA | No | No | ARDS  (O98·5,U07·1) | Pre-existing hypertension  (O10·9) |
| 21 | 20 | 3 MA | No | Yes | ARDS  (O98·5,U07·1) | Anemia  (O99·0) |
| 22 | 20 | 32 | Yes | No | ARDS  (O98·5,U07·1) | Pre-eclampsia (O14·1)  Anemia  (O99·0) |
| 23 | 24 | 39 | Yes | No | ARDS  (O98·5,U07·1) | Anemia  (O99·0) |
| 24 | 20 | 36 | Yes | No | ARDS  (O98·5,U07·1) | Anemia  (O99·0) |
| 25 | 25 | 34 | Yes | No | ARDS  (O98·5,U07·1) | Pre-eclampsia (O14·1)  Anemia  (O99·0) |
| 26 | 22 | 34 | Yes | - | ARDS  (O98·5,U07·1) | Anemia  (O99·0) |
| 27 | 20 | 36 | Yes | Yes | ARDS  (O98·5,U07·1) | Anemia  (O99·0) |
| 28 | 25 | 34 | Yes | - | ARDS  (O98·5,U07·1) | Pre-eclampsia (O14·1)  Anemia  (O99·0) |
| 29 | 24 | 3 MA | Yes | - | Sepsis  (O08·0) | Anemia  (O99·0)  COVID infection  U07·1 |
| 30 | 22 | PND | Yes | - | PPH  (O72·1) | COVID infection  (U07·1)  Anemia  (O99·0)  Peurperal Sepsis  (O85) |
| 31 | 25 | PND | Yes | - | Pre-eclampsia (O14·1) | COVID infection  (U07·1)  Anemia  (O99·0) |
| 32 | 27 | 2 MA | Yes | - | Abortion related hemorrhage  (O03·1) | COVID infection  (U07·1)  Anemia  (O99·0) |
| 33 | 23 | 40 | Yes | - | ARDS  (O98·5,U07·1) | Anemia  (O99·0) |
| 34 | 27 | PND-2 | Yes | - | PPH  (O72·1) | COVID infection  (U07·1)  Hypertension  (O13)  Anemia  (O99·0) |
| ARDS- Acute respiratory distress syndrome  ICU- Intensive care unit  MA-Months of amenorrhea  PND- Post natal day  PPH- Postpartum hemorrhage | | | | | | |
